# Management of asymptomatic sexually transmitted infections in Europe: towards a differentiated, evidence-based approach

**DOI:** 10.1016/j.lanepe.2023.100743

**Published:** 2023-10-26

**Authors:** Chris Kenyon, Björn Herrmann, Gwenda Hughes, Henry J.C. de Vries

**Affiliations:** a Department of Clinical Sciences, Institute of Tropical Medicine, Antwerp, Belgium; b Section of Clinical Microbiology, Department of Medical Sciences, Uppsala University, Uppsala, Sweden; c Department of Infectious Disease Epidemiology, London School of Hygiene & Tropical Medicine, UK; d Department of Dermatology, Amsterdam UMC Location University of Amsterdam, Meibergdreef 9, Amsterdam, the Netherlands; e Amsterdam Institute for Infection and Immunity, Infectious Diseases, Amsterdam, the Netherlands; f Center for Sexual Health, Department of Infectious Diseases, Public Health Service Amsterdam, the Netherlands; g Amsterdam Institute for Global Health and Development, Amsterdam, the Netherlands

## Abstract

Most sexually transmitted infections (STIs) can be accurately diagnosed and treated during asymptomatic carriage. Widespread screening for these STIs is therefore assumed to be an effective way to reduce their prevalence and associated disease. In this review, we provide evidence that this is the case for HIV and syphilis. However, for other STIs such as *Neisseria gonorrhoeae* and *Chlamydia trachomatis*, our review reveals that the evidence that screening reduces infection prevalence and associated disease is weak. There is also growing evidence of harms from screening that might outweigh any benefits. The harms include the increased consumption of antimicrobials that follows frequent screening and increased detection of asymptomatic STIs in key populations, such as men who have sex with men taking HIV pre-exposure prophylaxis, and associated risk of antimicrobial resistance in target and non-target organisms. There may also be psycho-social harm associated with an STI diagnosis. We conclude that in the absence of symptoms, in high STI prevalence populations frequent STI screening should be limited to HIV and syphilis.

**Key messages**

• Widespread screening is frequently assumed to be an effective way to reduce their prevalence and sequelae. The available evidence, however, supports a pathogen-specific approach to screening.
• HIV and syphilis are good candidates for screening, as both cause serious systemic infections and detectable serological responses. Early detection of these infections enables the initiation of life-saving treatment whilst preventing further transmission.
• However, for other STIs such as *Neisseria gonorrhoeae* and *Chlamydia trachomatis*, our review reveals that the evidence that screening reduces infection prevalence and associated disease is weak.
• There is also growing evidence of harms from screening that might outweigh any benefits, such as increased antimicrobial consumption following frequent screening and increased detection of asymptomatic STIs in key populations.
• This increased antimicrobial consumption may lead to antimicrobial resistance in target and non-target organisms.
• Screening for *N. gonorrhoeae*/*C. trachomatis* in HIV PrEP cohorts has been found to result in macrolide consumption that exceeds resistance inducing thresholds for various bacterial species by 5- to 9-fold.
• Excessive antimicrobial consumption may also exert deleterious effects on the microbiome, and a disrupted microbiome may offer less protection against STIs and other pathogens.
• There may also be psycho-social harms associated with the diagnosis of asymptomatic STIs. This may be particularly important in low STI prevalence populations where the positive predictive value of a test for *N. gonorrhoeae* may be as low as 17%–67%.
• International guidelines stipulate that screening programs should only be introduced once high-quality RCTs have established that the benefits of screening outweigh the harms, which is not available for *N. gonorrhoeae* or *C. trachomatis* in populations with a high STI prevalence.
• We conclude that in the absence of symptoms, frequent STI screening in high STI prevalence populations should be limited to HIV and syphilis.

## Background

**Fig. 1:**
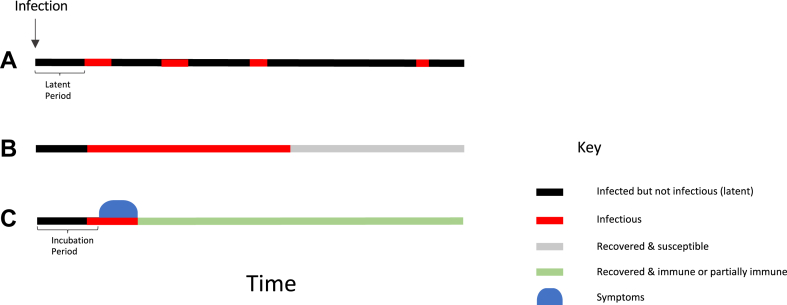
A schematic illustration of a number of ways that STIs can circulate asymptomatically in a population. (A) The majority of infections of certain STIs such as herpes simplex virus-2 (HSV-2) do not produce any symptoms. After a latent period, the HSV-2 does, however, become infectious (red line), before returning to latency in the dorsal root ganglia. It can then reactivate and become infectious again (with or without symptoms) for multiple periods for the following decades. (B) The vast majority of anorectal and pharyngeal *N. gonorrhoeae*, *C. trachomatis* and *M. genitalium* infections are asymptomatic and self-resolving. (C) For an STI such as mpox, the symptomatic and infectious periods are short, but the period of infectiousness begins before the symptomatic period (blue ovoid shape). Individuals can transmit the mpox virus in this presymptomatic period.

If a sexually transmitted infection (STI) produces severe symptoms or death this will likely impede its ability to be passed on to other people. This creates a selective pressure for STIs to produce few or no symptoms for long periods. Whilst this selection pressure may not apply to all STIs, the evolution of *Treponema pallidum* to diminished virulence in Europe in the 16th century may have resulted from this selective pressure.^1^ Within decades after its arrival in Europe, the clinical manifestations of syphilis were noted to be milder.^1^ These evolutionary pressures contribute to the asymptomatic nature of most STIs for most of the time they circulate in humans.^2^^,^^3^ We refer to an STI as ‘asymptomatic’ during the periods when it does not cause any discernable symptoms. This includes the incubation period before symptoms emerge and the period following symptom resolution whilst the infection is still present (Fig. 1). Some STIs are asymptomatic for the entire duration of infection.

Most STIs can be accurately diagnosed and treated during asymptomatic carriage. Widespread screening for these STIs is therefore assumed to be an effective way to reduce their prevalence and associated harms. There is indeed compelling evidence that this is the case for HIV and syphilis. However, for other STIs such as *Neisseria gonorrhoeae* (NG) and *Chlamydia trachomatis* (CT) there is growing evidence that the harms of screening may outweigh any benefits. The harms of screening are often related to the increased antimicrobial consumption following increased detection of asymptomatic STIs, and the psycho-social harms of an STI diagnosis.^4^ At the same time, the increasing incidence of many STIs primarily in high-income countries has led to calls for more screening for asymptomatic STIs.^5^ In this review paper, we summarize the microbiological, clinical and epidemiological literature showing the frequency of asymptomatic STIs. We review the evidence of the harms and benefits of screening and present emerging evidence that supports a pathogen-specific, differentiated approach to screening. International guidelines stipulate that screening programs should only be introduced once they have met specific criteria. These criteria include the need for evidence from high-quality RCTs that screening is effective in reducing mortality or morbidity, the benefits should outweigh the harms and screening should be cost effective.^6^ In this article, we review the evidence from RCTs and other types of studies and argue that the available evidence favours intensive screening for STIs such as syphilis and HIV in certain populations. For other STIs such as NG and CT the evidence does not support frequent (3 monthly) screening i.e. testing of asymptomatic individuals on the basis of sexual behaviour.

**Search strategy and selection criteria**

References for this review were identified through searches of PubMed and GoogleScholar with the search terms “asymptomatic”, “STI”, “chlamydia”, “gonorr∗”, “syphilis”, and “screening” from 1980 until May, 2023. Articles were also identified through searches of the authors’ own files. Only papers published in English were reviewed.

## Most STIs are asymptomatic

For many bacterial STIs, the majority of infections are asymptomatic. In the case of CT, only 11%–33% infections in men and 6%–17% in women become symptomatic.^2^^,^^3^^,^^7^ The corresponding figures for symptomatic NG are 45%–85% in men and 14%–35% in women.^2^^,^^3^^,^^7^ The presentation of syphilis is more complex.^8^ Most people infected with *T. pallidum* develop symptoms the first time they are infected.^9^^,^^10^ These symptoms are however frequently not diagnosed as syphilis as the chancre of primary syphilis may be located in inconspicuous sites or body orifices such as the anorectum, vagina, urethra, oropharynx or hand where they may not be noticed or recognized.^8^ In addition, people with multiple previous episodes of syphilis are considerably less likely to develop symptoms of syphilis.^11^^,^^12^

Most people with viral STIs such as herpes simplex virus-2 (HSV-2) and human papilloma virus are asymptomatic.^2^ For example, in a prospective study of HSV-2-seronegative persons, of the 155 who acquired HSV-2, only 57 (37%) developed symptoms during the observation period.^13^ Likewise, serological surveys among the general population have shown that nearly all people with antibodies to HSV-2 have never had symptoms of genital herpes.^14^ Longitudinal couple studies have however established that most HSV-2 transmissions occur when the index patient is asymptomatic.^15^

A recent large outbreak of mpox, caused by subclade IIb of the mpox virus that involved over 80,000 cases was predominantly spread via sexual contact.^16^ Cases typically had cutaneous lesions suggestive of a pox virus infection as well as systemic symptoms including proctitis and pharyngitis.^16^ For some time, it was unclear how this virus continued to spread so widely if affected populations abstained from sex once they had symptoms and were infectious.^17^ Various types of evidence, including prospective couple transmission studies have subsequently demonstrated that a sizable of proportion transmissions occurred whilst the index was asymptomatic or had atypical symptoms.^16^^,^^18^^,^^19^

## Syphilis and HIV—good candidates for STI screening

The development of the non-treponemal tests (such as the Wassermann Reaction, RPR and VDRL) were the first serological tests used in medicine.^20^ These tests revolutionized the approach to syphilis as they provided a relatively accurate way to confirm the diagnosis of syphilis, assess the response to therapy and ascertain if individuals were asymptomatically infected.^10^ As an example of widespread screening, by 1954 all but 8 states in the United States required a negative syphilis test before individuals could get married.^21^ It is unclear what impact this widespread testing had on the spread of syphilis but the widespread introduction of testing and treatment with highly efficacious penicillin is widely believed to have played a crucial role in the dramatic decline of syphilis prevalence in many regions around the world in the post-World War II period.^22^

If we consider the host-pathogen interactions of *T. pallidum*, it has a number of attributes which make it a good candidate for screening (Table 1).^23^ First, it has a long incubation period of up to 3/6 months for primary/secondary syphilis and years to decades before symptoms of late-stage syphilis develop.^8^ This offers a long period in which to detect the infection prior to the onset of the late-stage disease. Second, the late-stage manifestations include severe irreversible disease such as dementia, stroke, blindness and ruptured aortic aneurysms.^8^^,^^10^^,^^24^ Third, we have accurate diagnostic tests.^25^ Fourth, penicillin is a highly efficacious treatment which can prevent late-stage disease if given early enough.^10^ Fifth, penicillin treatment confers a relatively low risk of inducing antimicrobial resistance (AMR) in other bacteria and penicillin-resistance has never been described in *T. pallidum*.^8^^,^^23^ Sixth, as already noted, people with previous episodes of syphilis are less likely to develop symptoms of syphilis.^11^^,^^12^ This means that their syphilis reinfections will likely be missed unless they are screened sufficiently frequently. Screening people with previous episodes of syphilis thus becomes vital to prevent them from: 1) developing late manifestations of syphilis with potential irreversible cardiac and neurological damage, and 2) onward transmission to their partners.^11^ Screening for syphilis is thus vital in persons at increased risk of exposure based on epidemiological grounds, such as men who have sex with men (MSM) who use Pre-Exposure Prophylaxis (PrEP) against HIV infections, and pregnant women to prevent mother to child transmission (MTCT) and subsequent detrimental pregnancy-related outcomes and neo- and perinatal death.^8^^,^^26^

**Table 1:** Non-exclusive list of possible criteria for evaluating net utility of screening six specific STIs in MSM PrEP cohort^a^.^23^

	*N. gonorrhoeae*	*C. trachomatis*	*M. genitalium*	*T. pallidum*	HIV
**Are host-pathogen interactions amenable to screening?**					
1. Undetected infection typically associated with serious adverse clinical outcomes	+	+	−	+++	+++++
2. Long period between infection and disease onset	−	−	−	++	+++
3. Not spontaneously cleared by immune system	−	−	−	+++	+++++
4. Natural immunity from recovered infection	+++	+	+++	+	++++
**High risk of inducing AMR?**					
1. High risk of inducing AMR in pathogen itself given standard therapy	++++	+	++++	+	−
2. High risk of inducing AMR in microbiome given standard therapy	+++	++	+++	+	

Example 1. For the first criterion, there is little or no evidence that MG is associated with serious adverse clinical outcomes and MG is thus scored ‘−’, whereas there is plenty of evidence that HIV is associated with severe outcomes and HIV is thus scored ‘+++++’.

Example 2. In the case of HIV for the fourth criterion, an HIV infection is not eradicated by the immune system and thus there is no immunity. HIV thus gets a favourable score for being amenable to screening on this criterion.

a This scoring is not based on a systematic review but on a subjective assessment of the authors’ evaluation of the scientific literature. Each infection is rated from ‘−’ to ‘+++++’ according to the evidence base underpinning the criterion and the clinical significance.

These attributes mean that screening individuals at risk for syphilis results in the detection and treatment of infections before they cause severe morbidity and with a low risk of inducing AMR. By reducing the duration of infection, screening and early treatment would also be expected to reduce the spread of syphilis.^8^^,^^27^^,^^28^

Similar arguments apply to screening for HIV (Table 1). In contrast to NG, CT and MG which produce mucosal infections, HIV and syphilis cause systemic infections and as a result, detectable serological responses. Both infections can therefore be detected early after infection via accurate serological tests.^29^ Early detection opens the way to both lifesaving therapy and reducing the onward transmission of the infection.^29^^,^^30^ This in turn can contribute to the local elimination of these infections.^4^ In the case of HIV in the Netherlands, the test and treat strategy has contributed to an estimated 70% reduction in incidence in the past 10 years.^4^

## *N. gonorrhoeae*, *C. trachomatis* and *M. genitalium*—poorer candidates for screening

### Weak evidence that screening reduces harm

**Fig. 2:**
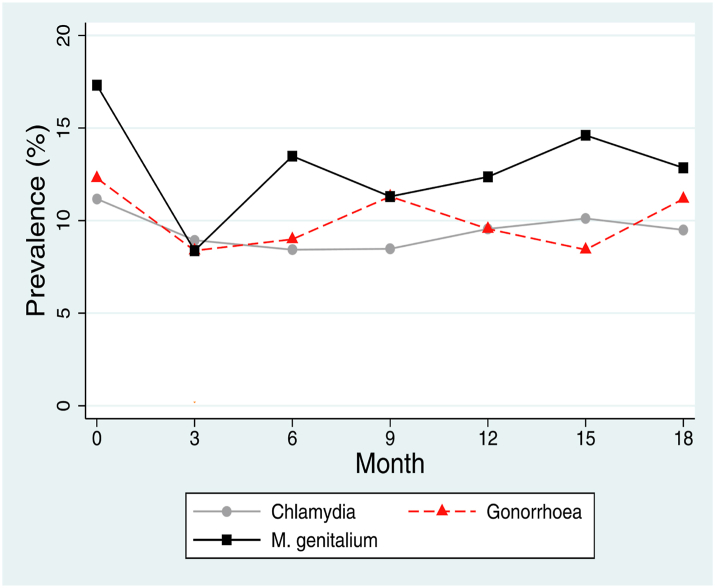
Prevalence of *N. gonorrhoeae*, *C. trachomatis* and *M. genitalium* in a typical PrEP cohort in Belgium. Participants were screened at 3-sites (pharynx, anorectum and urethra) every three months for each of these STIs.

In contrast to syphilis, the host pathogen interactions of NG, CT, and MG might make them less amenable to screening (Table 1). Whereas syphilis and HIV are systemic infections, NG, CT, and MG are typically self-limiting, mucosal infections and have similar host-pathogen interactions (Table 1, Fig. 2).

#### Frequent screening in key populations

If we consider the example of NG circulating in MSM, the vast majority of infections are asymptomatic and self-limiting (cleared by the immune system).^3^^,^^31^^,^^32^ The incubation period of NG is short (2–21 days) and after this period, the infection persists in a low abundance state for up to 6 months until it is eradicated by the immune system or interactions with the hosts microbiome.^33^ Intensive screening among multi-partner MSM may be more likely to detect infections in the 6-month asymptomatic tail phase (when NG abundance is likely lower and therefore less infectious) than in the acute first 21 days post infection.^23^ These features reduce the probability that screening will reduce the incidence of symptomatic infections and complications. If the low abundance chronic tail of NG infections are less infectious than the early period of infection, then this will further reduce the probability that screening will interrupt the transmission of NG. In the small proportion of infections that become symptomatic, these can be treated with rapid resolution of symptoms.^33^

#### General population screening

Infection with NG, CT and to a lesser extent MG have been causally associated with PID and tubal infertility in women.^34^^,^^35^ An evidence synthesis estimated that the risk of ectopic pregnancy and tubal infertility following an untreated CT infection was 0.2% and 0.5%, respectively,^36^ and estimated that 29% of tubal infertility was due to CT. However, it does not necessarily follow that screening for CT will reduce the incidence of these outcomes.^4^ A systematic review of RCTs found low to moderate quality evidence that screening CT was associated with a reduced incidence of PID.^37^ The studies at lower risk of bias did however reveal less of a reduction in PID than those at higher risk of bias.^37^ The importance of basing policy on RCT based evidence of adverse outcomes is illustrated by the example of bacterial vaginosis. Bacterial vaginosis is causally associated with PID and various adverse pregnancy outcomes. However, various screening RCTs have established that screening for bacterial vaginosis is not associated with improvement in these outcomes.^38^

A working group of Dutch national and international experts reviewed this and related data in 2021 and concluded that the available evidence supported reducing rather than expanding chlamydia testing.^4^ One of their arguments was that PID can be easily treated once symptoms develop, that one major reason to screen women is to prevent tubal infertility and that they were unable to find evidence that screening has this effect. The damage done by CT may be done early on in the infection before the infection is detected and treated so the fraction of adverse pregnancy outcomes that could be prevented by chlamydia screening is therefore unknown.^4^ For example, one study that followed women with and without chlamydia infection found no difference in the proportion getting pregnant.^39^ These findings suggest that the net benefit of screening for CT on key outcomes such as tubal infertility may be small or zero.^4^^,^^36^ Following a review of the evidence by national and international experts commissioned by the National Chlamydia Screening Programme in England, the programme acknowledged that uncertainty exists about the amount of harm prevented and recommended more focussed testing at partner change and through partner notification.^40^

### Weak or no evidence that screening reduces prevalence

Two large cluster RCTs have evaluated the effect of screening for CT in general populations^41^^,^^42^ and both found no significant impact of screening on CT prevalence. No RCTs have been conducted to evaluate the efficacy of screening for NG in general populations.^43^

Likewise, a systematic review of observational studies of the effect of screening on the prevalence of NG and CT in MSM found that the intensity of screening had no detectable effect on the prevalence of these infections.^43^ This is illustrated in Fig. 2, which depicts the prevalence of NG, CT and MG in a typical PrEP cohort. Despite very intense screening (testing oropharynx, rectum and urine every three months) and treatment, the prevalence of these three infections was not reduced.^44^ These findings led first to the cessation of screening for MG and subsequently reduction of NG/CT screening intensity from 3 anatomical sites per 3 months to one site per 6 months. The cessation of MG screening led to a 38- and 2-fold decline in fluoroquinolone and macrolide consumption respectively but no appreciable increase in symptomatic or asymptomatic MG infections over the subsequent 12 months.^45^ Similarly, reducing NG/CT screening resulted in an additional 6-fold reduction in macrolide exposure without any noticeable effect on numbers of symptomatic infections.^46^

Ecological analyses have found that countries where MSM are more intensely screened for NG/CT do not have a lower incidence and prevalence of asymptomatic or symptomatic NG/CT cases.^47^ Further evidence comes from a large study of self-reported data from two surveys in 2010 and 2017 of over 100,000 MSM from 46 European countries. The study found that the intensity of NG/CT screening increased over time, but the intensity of screening was positively associated with the number of symptomatic NG/CT cases.^47^

**Fig. 3:**
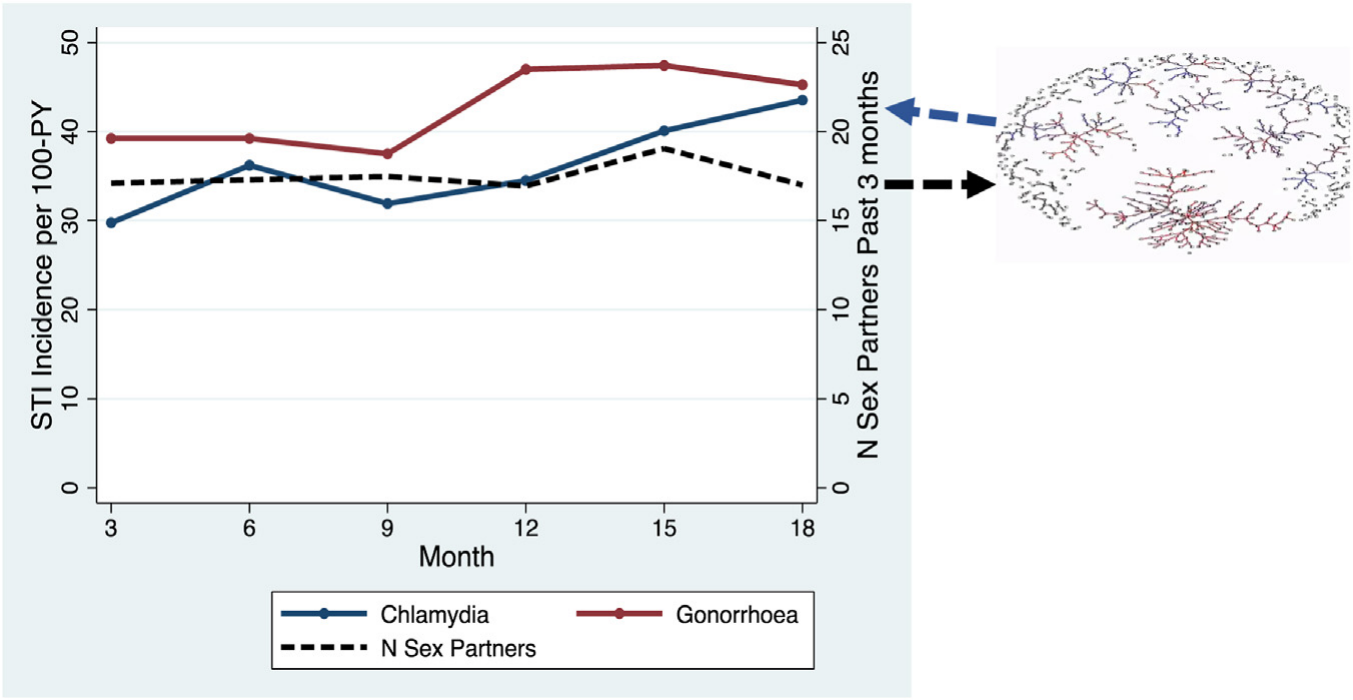
An illustration of the connection between sexual network connectivity and equilibrium prevalence of *N. gonorrhoeae* and *C. trachomatis* using data from the ANRS-Prevenir study of PrEP in France.^51^ The relatively high rate of partner turnover (15–20 partners per 3 months) generates a dense sexual network (black arrows), which in turn, sustains a high equilibrium prevalence/incidence (blue arrows) of both *N. gonorrhoeae* and *C. trachomatis* (between 30 and 50 infections per 100 person years).

How can we explain the fact that intense screening for CT, NG and MG does not appear to reduce the prevalence of these infections in MSM on PrEP? A key factor appears to be the dense sexual network of these populations.^44^^,^^48^, ^49^, ^50^ This is illustrated in Fig. 3, where participants report a steady average of 15–20 partners per three-month period. Modelling studies have shown that this translates into a dense sexual network which would generate an equilibrium prevalence of around 10%–15% for CT, NG and MG.^49^^,^^50^^,^^52^ Randomly selecting individuals from this network for screening and treating their asymptomatic NG may do little to reduce NG prevalence as it has no impact on the underlying determinant of high equilibrium prevalence—the dense sexual network.^48^^,^^49^ Whilst, very intense screening, such as weekly screening, may lead to reduced NG prevalence, this would be at the expense of even greater antibiotic exposure.^49^ Furthermore, this reduced prevalence of NG would create an evolutionary pressure for NG to return to its equilibrium prevalence.^48^ One way to achieve this would be for circulating NG to acquire resistance to the antimicrobials used to suppress its equilibrium prevalence.^48^ This leads to the paradoxical conclusion that reducing the prevalence of STIs such as NG and MG in populations with dense sexual networks may not be desirable as it may select for AMR in these and other non-target species.^44^^,^^53^ Of course, if the screening was sufficiently intense to eradicate the infection, then this risk of AMR would be less likely to apply.

### Screening and treatment for NG/CT/MG results in adverse effects on the microbiome and resistome

Studies have confirmed that the high equilibrium prevalence of NG/CT in PrEP cohorts means that screening MSM for these STIs results in high levels of antimicrobial consumption.^46^ For instance, three-site, three-monthly NG/CT screening results in up to 12 defined daily doses of macrolides per 1000 inhabitants per year (DID).^46^ Twelve DIDs exceeds the approximate thresholds for the induction of macrolide resistance in *Mycoplasma genitalium*, *Streptococcus pneumoniae* and *T. pallidum* by 5- to 9-fold.^54^ Increased antimicrobial consumption is of particular concern in MSM with high rates of partner change such as those taking PrEP, as gonococcal AMR has frequently emerged in groups heavily exposed to antimicrobials.^55^ An ecological study has also found a positive association between the intensity of screening MSM for NG/CT and reduced gonococcal susceptibilities to cephalosporins,^56^ while another using linked historical records has shown that repeat diagnosis of gonorrhoea through quarterly screening in MSM may be associated with reduced ceftriaxone and cefixime susceptibility.^57^

Individual level studies have revealed the detrimental effects of antimicrobials such as ceftriaxone and azithromycin on both the microbiome and resistome. A single dose of azithromycin, for example, has been shown to reduce the recipients’ colonic microbial diversity for 6 months as well as increasing the proportion of oral streptococci with macrolide resistance for over 6 months.^58^, ^59^, ^60^ Short courses of macrolides have also been shown to result in elevated macrolide resistance genes in the colon for 4 years.^61^ The receipt of ceftriaxone and azithromycin have also been linked to reduced susceptibility to these antimicrobials in commensal *Neisseria* species.^62^^,^^63^ This is important as gonococcal uptake of resistance conferring DNA from these commensal *Neisseria*, has played a crucial role in the emergence of gonococcal resistance to both ceftriaxone and azithromycin.^64^^,^^65^ Excess exposure to antimicrobials is also one of the plausible explanations for the emergence of a number outbreaks of antibiotic resistant enteric pathogens in MSM.^53^

Antimicrobial induced dysbioses have also been linked to an increased incidence in a range of adverse clinical outcomes such as atopy, asthma, obesity and Chron’s disease.^66^^,^^67^

A healthy, diverse microbiome has also been shown to provide colonization resistance to pathogens.^68^ Studies have established that broad-spectrum antimicrobials such as aminoglycosides and third generation cephalosporins lower the colonization resistance (number of colonies of a pathogen required to establish a symptomatic infection) by up to 10,000-fold.^68^ Certain commensal *Neisseria* and streptococcal species have been shown to be able to protect against gonococcal infection.^69^^,^^70^ As noted above, intensive screening for STIs such as NG, CT and MG can result in considerable antimicrobial exposure. This has in turn been shown to adversely affect the composition of commensal *Neisseria* species which may increase susceptibility to subsequent NG infections.^71^^,^^72^ Likewise, a mouthwash-induced alteration in commensal bacteria is one of the most plausible explanations for the unexpected results from an RCT which found that the daily and peri-sexual use of an antiseptic mouthwash increased rather than reduced the incidence of pharyngeal NG.^73^ An additional concern is the toxicity of some of the second-line therapies for MG. Increasing macrolide resistance has led to increasing use of fluoroquinolones such as moxifloxacin which have received FDA boxed warnings pertaining to side effects.^74^

## Screening expands to other ‘STIs’ in an era of multiplex-PCRs

The success of screening strategies for syphilis and HIV has been followed by a rapidly expanding list of ‘STIs’ that may be screened for, often without the backup of professional guidelines.^44^ As an example, one multiplex PCR in widespread use in Belgium, tests a panel of 17 viral, bacterial and protozoan ‘STIs’, that includes *Mycoplasma hominis* and *Ureaplasma parvum*.^44^ Although *M. hominis* and *U. parvum* may be sexually acquired, they are not considered pathogenic, and various guidelines argue that these organisms should be considered part of the ‘normal’ genital microbiome.^75^ Eradicating these organisms with antimicrobial medications is often not feasible and may result in microbiome disruption. The high prevalence of these organisms in adult women (5%–20% for *U. parvum* and 20%–89% for *M. hominis*),^75^ means that a majority of women may be diagnosed with an ‘STI’ every time they are tested with one of these multiplex tests.^44^ Receiving the diagnosis of an ‘STI’ can result in adverse psychological consequences.^75^^,^^76^

The appreciation of these dangers of screening for these infections led the International Union against STI (IUSTI) European STI Guidelines Editorial Board to recommend against routine screening or testing for *U. parvum* and *M. hominis* in STI clinics.^75^ Likewise guidelines for *M. genitalium* clearly state that testing should be focused on symptomatic individuals and that the available evidence does not support screening.^77^^,^^78^ There is now widespread agreement that asymptomatic individuals should not be screened for *M. genitalium*.^75^^,^^77^^,^^78^

Combination testing for *C. trachomatis* and *N. gonorrhoeae* is commonly used for the major commercial test systems. The impact of this on the prevalence of *N. gonorrhoeae* in low prevalence populations is largely unknown. However, a study in England of dual CT/NG screening in the English National Chlamydia Screening Programme estimated that the positive predictive value of NG screening was between 17% and 67%, indicating high risk of false-positive test results and potentially unnecessary distress caused to those receiving reactive results.^79^

## Variations in opinions and guidelines towards screening

It is important to acknowledge the wide range of opinions about which STIs should be screened for, in which populations and how frequently. In part, this diversity is related to the weak evidence base of the benefits and harms of screening for each of the STIs across diverse populations and is reflected in differences in national screening guidelines.^80^ Whilst the expert panel convened in the Netherlands recommended less intense or no chlamydia screening among young heterosexual adults,^4^ other panels such as the United States Preventive Services Task Force (USPSTF) continue to recommend chlamydia screening in all sexually active women below 25 years old.^81^ The USPSTF has however reduced the strength of this recommendation from ‘A’ to ‘B’.^81^ There are also large differences in chlamydia screening guidelines between European countries.^80^

These divergences suggest the need for additional clinical trials to compare the full range of benefits and risks of screening for chlamydia and other STIs in different population groups. In particular, very few STI screening RCTs have been conducted in low and middle-income countries and extrapolating study results from high- to low-and-middle-income-countries requires caution. For example, two large chlamydia screening RCTs found that screening was not associated with a reduced prevalence of CT.^41^^,^^42^ These RCTs were, however, performed in general populations in high income countries with a low CT prevalence. RCTs conducted in populations with higher network connectivity and chlamydia prevalence, including some in Southern Africa, might show an impact of screening on CT prevalence.^82^^,^^83^ It is, however, also possible that screening could reduce immunity and thereby lead to a rebound increased CT prevalence as well as increased AMR due to the increased antimicrobial consumption.^84^^,^^85^ RCTs in these populations are thus urgently required to assess the net harms and benefits of screening.^86^ Similarly, RCTs might help to evaluate the efficacy of screening for NG/CT in MSM. The results of the first RCT exploring the impact of frequent screening on NG and CT incidence in MSM are expected to be published soon.

## Conclusion

As already noted, international guidelines stipulate that screening programs should only be introduced once high-quality RCTs have proven their net effectiveness.^6^ Our appraisal of the literature is that for CT, NG and MG the evidence is weak. On the one hand, there is some evidence that CT screening may reduce the incidence of PID in general populations but the possible benefit this will extend to preventing tubal infertility is unclear.^4^^,^^36^ On the other hand, there is no robust evidence on the benefits of frequent screening for NG, CT and MG in MSM, and increasing evidence of the harms of screening for these infections, particularly in populations like subpopulations of MSM with dense sexual networks. In these populations screening has been shown to result in antimicrobial consumption of broad-spectrum antimicrobials considerably in excess of resistance inducing thresholds. In addition to selecting for AMR in pathogens and commensals, this intense exposure may result in dysbiosis, arrested immunity and reduced colonization resistance.^84^^,^^85^ These negative effects are clearest for MG where numerous authors explicitly recommend restricting the testing of asymptomatic individuals for MG.^77^^,^^87^

In contrast, we have good evidence to advocate screening for HIV and syphilis in populations at greatest risk, including those in dense sexual networks such as MSM on PrEP. These divergent findings provide the basis for our recommendation of a population and pathogen-specific approach to STI screening. It is likely that the risk, benefit and cost-effectiveness of screening will vary according to the population and pathogen tested, diagnostic test used, testing frequency and the health system model. The preferences of the population-served and the individual client should also play an important role in determining our approach to screening. Future research will be necessary to better delineate in which circumstances it is appropriate to promote screening for each STI. The results of a number of screening RCTs are awaited with great anticipation.^86^^,^^88^

## Contributors

CK performed the literature review and wrote a first draft of the paper. BH, GH and HdV all provided extensive input into subsequent versions of the paper.

## Declaration of interests

All authors declare that they have no conflicts of interest. GH is the PI at London School of Hygiene and Tropical Medicine (LSHTM) on funding for the UK Public Health Rapid Support Team, which is funded by Official Development Assistance (ODA) via the Department of Health and Social Care & the National Institute for Health Research (NIHR) Central Commissions Facility.
